# Beta2 Adrenergic Receptor (*ADRβ2*) Haplotype Pair (2/4) Is Associated with Severe Asthma

**DOI:** 10.1371/journal.pone.0093695

**Published:** 2014-04-01

**Authors:** Li Ping Chung, Svetlana Baltic, Manuel Ferreira, Suzanna Temple, Grant Waterer, Philip J. Thompson

**Affiliations:** 1 Molecular Genetics and Inflammation Unit, Lung Institute of Western Australia and Centre for Asthma, Allergy and Respiratory Research, University of Western Australia, Crawley, Nedlands, Western Australia, Australia; 2 School of Medicine and Pharmacology, University of Western Australia, Crawley, Western Australia, Australia; 3 The Queensland Institute of Medical Research, Brisbane, Queensland, Australia; 4 Department of Respiratory Medicine, Royal Perth Hospital, Perth, Western Australia, Australia; 5 Department of Respiratory Medicine, Sir Charles Gairdner Hospital, Nedlands, Western Australia, Australia; Cincinnati Children's Hospital Medical center, United States of America

## Abstract

**Background:**

*β2* adrenergic receptor (*ADRβ2*) polymorphisms including *ADRβ2*+46G>A have been reported to cause adverse outcomes in mild asthmatics. The extent to which *ADRβ2* polymorphisms and in particular their haplotypes contribute to severe asthma is unknown.

**Objective:**

To determine the association of *ADRβ2* polymorphisms and haplotypes with asthma severity.

**Methods:**

Caucasians (n = 2979) were genotyped for 11 *ADRβ2* polymorphisms. The cohort (mean age 39.6, 60% female) included 2296 non-asthmatics, 386 mild asthmatics, 172 moderate asthmatics and 125 severe asthmatics. Haplotype frequency and haplotype pair for each subject was determined using the PHASE algorithm.

**Results:**

The three asthmatic cohorts were comparable in age and gender but were distinguishable from each other in terms of symptoms, spirometry, medication use and health care utilisation (p <0.001). None of the polymorphisms showed a genotypic or allelic association with asthma diagnosis or severity. Nine haplotypes were identified and no association was found with asthma diagnosis or severity *per se*. Haplotype pair 2/4 was associated with asthma severity (Trend Test, OR 1.42, p = 0.0008) but not with asthma *per se*. Prevalence of haplotype pair 2/2 appeared to decrease with asthma severity (Trend Test, OR 0.78, p = 0.067). Two new haplotypes were identified, occurring exclusively in asthmatics at a frequency of ≥ 1%. In addition, a positive association between carriage of *ADRβ2* +523*C and increased risk of atopy was discovered.

**Conclusions:**

*ADRβ2* haplotype pair 2/4 is associated with severe asthma and is consistent with findings of poor bronchodilator response in mild asthmatics who are also haplotype 2/4.

## Background

Asthma is a common inflammatory airways disease affecting 12% of adults in the Australian population. Approximately 10% of these have severe persistent disease and remains poorly controlled despite maximal therapy. Response to medications varies significantly between individuals. Although medication adherence is a significant issue, it is estimated that up to 70% of variability in therapeutic responses to pharmacotherapy is genetically based.[1]–[3]

β2 adrenergic receptor (*ADRβ2*) agonists are the most commonly prescribed and used inhaled asthma therapy. Their efficacy is partially dependent upon their molecular conformation and properties and the density, structure and conformation of the *ADRβ2* on the cell surface. The expression/regulation of *ADRβ2* is altered by several single nucleotide polymorphisms (SNP) within the promoter, coding and the 3′UTR domains. [4]–[6] *ADRβ2*+46*G (Gly16) results in increased agonist-stimulated receptor down-regulation while *ADRβ2*+79*G (Glu27) is highly resistant.[7] *ADRβ2*+491*T (Ile164) alters receptor binding affinity with reduced activation *in vitro* [8] and blunted *in vivo* responses to terbutaline in healthy and cardiac failure subjects. [9], [10]

Clinical studies in mild asthmatics have shown differential clinical response attributable to genetic variation. *ADRβ2*+46*A homozygotes are more likely to bronchodilate with salbutamol compared to individuals homozygous for the G allele.[11], [12] In contrast, prolonged or frequent use of short or long acting *ADRβ2* agonist (SABA, LABA respectively) in mild asthmatics homozygous for *ADRβ2*+46*A is associated with adverse outcomes.[13]–[17]

Larger pharmacogenetic studies on *ADRβ2* polymorphisms and asthma have almost exclusively involved those with mild or moderate disease while the significance of genetic variation in severe asthma remains unclear.[18]–[21] Three studies that examined the relationship between *ADRβ2* polymorphisms and severe asthma produced contradictory results.[22]–[24] These include an association between severe asthma and *ADRβ2*+79*G (odds ratio 1.91) [22]; a fall in FEV_1_ and tachyphylaxis with formoterol in *ADRβ2*+46*G homozygotes [23]; and no association between *ADRβ2*+46 or +79 genotype in fatal or near fatal asthma.[24] However, severe asthma is a complex disease and it is unlikely explained by single genotypes (*ADRβ2*+46 and +79) *per se* but rather requires analysis of rarer variants and haplotypes.

**Figure 1:**
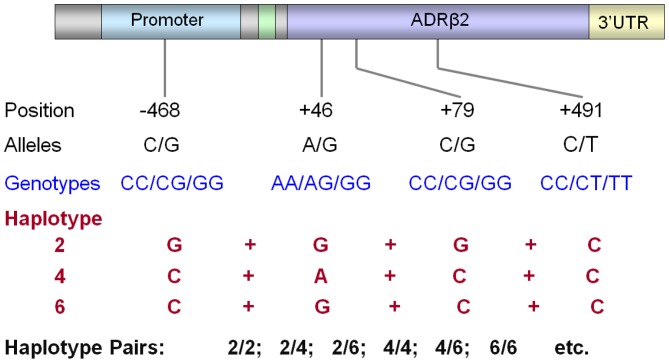
Schematic representation of *ADRβ2* genotype, haplotype and haplotype pair.

Linkage disequilibrium of the *ADRβ2* gene dictates that alleles at different locations of the *ADRβ2* gene with opposing *in-vitro* effects are commonly co-inherited as a haplotype and the outcome of their interaction is hard to predict. Receptors with *ADRβ2*+79*G polymorphism are highly resistant to agonist-induced downregulation *in vitro* yet co-inheritance of *ADRβ2*+46*G favours *ADRβ2*+46*G induced down-regulation.[25] It is therefore critical that *ADRβ2* haplotypes are assessed in addition to individual genotypes.[26] Each individual inherits two copies of the *ADRβ2* gene derived from the maternal and paternal copy of chromosome 5. This in effect means that *ADRβ2* gene is inherited, not simply as genotypes or single haplotype, but as a haplotype pair (Figure 1) which makes it even more difficult to ascertain the functional and clinical significance of *ADRβ2* polymorphisms.

We hypothesized that *ADRβ2* polymorphisms or haplotypes associated with altered receptor expression/regulation are more prevalent in severe asthma. As such our aim was to determine the frequency of selected SNPs and identify and compare the common haplotypes and haplotype pairs in mild, moderate and severe asthma and with healthy individuals.

## Methods

### Overview of Study Design

Asthmatics and healthy controls were recruited from the Lung Institute of Western Australia and the Queensland Institute of Medical Research. All participants were extensively genotyped for the *ADRβ2* gene. Statistical analyses were performed to determine the relationship between *ADRβ2* gene and asthma severity and presence of atopy by looking at the frequency of various genotypes, haplotypes as well as haplotype pairs in the relevant patients groups.

### Subject Recruitment

**Lung Institute of Western Australia (LIWA) Asthma Genetic Cohort.** Non-asthmatic controls (n = 200) were recruited through contacting individuals randomly selected from the metropolitan telephone directory. Mild, moderate and severe asthmatic cohorts were similarly recruited but enriched by recruiting from specialty asthma clinics and hospital databases.

Asthma (n = 683) was defined as having a respiratory physician-diagnosis of asthma based upon clinical history, spirometry, salbutamol reversibility and/or positive methacholine challenge tests. Mild and moderate asthma were defined as published previously using a number of criteria including lung function (FEV_1_ % predicted, use of oral steroids in the last 12 months, daily inhaled corticosteroid dose, weekly frequency of use of rescue medication, weekly frequency of daytime symptoms, weekly frequency of nocturnal awakening due to asthma, unplanned visits to doctor in the last 12 months and hospital admissions for asthma in the last 12 months.[27], [28] Patients were classified as having mild asthma if they have normal lung function (FEV_1_ greater than 80% predicted), frequency of daytime or night-time symptoms and use of rescue medication use less than twice per week with no unplanned medical visits or hospitalisations in the previous 12 months while treated with low dose inhaled corticosteroid therapy. Severe refractory asthma patients had to meet the American Thoracic Society (ATS) Criteria [29] and to have experienced at least 2 asthma-related hospital admissions within any 12 month period between January 2003 and February 2010. Patients with symptoms and treatment intermediate to mild and severe classification were labelled as moderate asthmatics.

All participants completed a questionnaire which included an in depth description of their symptoms and medication use. They were reviewed by a respiratory physician who confirmed their diagnosis and severity. All participants (including healthy controls) underwent spirometry in accordance with the ATS guidelines as well as allergen skin prick testing to 5 common aeroallergens: cat, dog, house dust mite, mould mix and grass pollen mix. Atopy was defined as positive skin reaction (wheal diameter ≥ 3 mm) to at least one allergen. The study was approved by the Human Research Ethics Committees at Sir Charles Gairdner and Royal Perth Hospitals. All subjects provided written consent to all study procedures including allergy and genetic testing.

#### Queensland Institute of Medical Research (QIMR) Non-Asthmatics from Genome Wide Association Study (GWAS)

To improve the power to detect a true genetic association, we expanded the non-asthmatic LIWA cohort to include 2096 unrelated Caucasians ascertained by QIMR and included in a recent GWAS of asthma.[30] Of these, 1248 were considered asthma-free based on a negative response to the questions “How often have you had asthma?”, “Have you ever had asthma?”, “Has a doctor ever diagnosed you as suffering from asthma?”. There was no information available on the asthma status of the remaining 848 individuals, but these were nonetheless included as unselected controls to improve power. There were no significant differences in SNP allele frequencies between the QIMR and LIWA control groups, thus supporting our decision to merge both datasets.

### Genotyping and Determination of *ADRβ2* Haplotype and Haplotype Pairs

Genomic DNA was extracted from buffy coat by QIAamp DNA Blood Mini Kit (Qiagen, Hilden, Germany). A total of 11 SNPs of the *ADRβ2* gene (Table 1) were genotyped using Taqman Genotying Assay (Applied Biosystems, Foster City, CA, USA). Unequivocal results from Taqman Assays were confirmed by PCR and direct sequencing. The QIMR_610 k cohort was genotyped using the Illumina 610K Assay (Illumina, San Diego, CA, USA) as described previously.[30] A subgroup of the LIWA cohort were also included in the GWAS (n = 645), thereby allowing quality control assessment of *ADRβ2* genotyping by both genotyping techniques. The QIMR_610k cohort has been genotyped for numerous other genes related to asthma and atopy and these results are not yet available for public access but may be shared upon request.

**Table 1:** *ADRβ2* Polymorphisms Genotyped.§

Polymorphisms*	dbSNP rs No.	Location	Alleles	Amino Acid Change	*In Vitro* Effect
−1023	rs 2053044	5′UTR	G/A		
−654	rs 12654778	5′UTR	G/A		
−468	rs 11168070	5′UTR	C/G	His^174^Asp	
−367	rs 11959427	5′UTR	C/T	Pro^207^Pro	Lower receptor expression with C allele[53]
−47	rs 1042711	BUP	T/C	Cys > Arg	Lower receptor expression with C allele [54]
−20	rs 1801704	5′ UTR	T/C		
+46	rs 1042713	Coding	G/A	Gly^16^Arg	Reduced receptor downregulation with A allele (Arg16)[7]
+79	rs 1042714	Coding	C/G	Gln^27^Glu	Reduced receptor downregulation with G allele (Glu27)[7]
+252	rs 1042717	Coding	G/A	Leu^84^Leu	
+491	rs 1800888	Coding	C/T	Thr^164^Ile	Reduced agonist binding affinity with T allele (Ile164)[8]
+523	rs 1042718	Coding	C/A	Arg^175^Arg	

Definition of abbreviations: BUP  =  β upstream peptide, also known as 5′leader cistron.

*Position of SNP relative to ATG start codon for *ADRβ2* gene.

§ Selected based on known functional effects on receptor modulation and those required for accurate characterisation of haplotypes previously defined by Drysdale *et al.* [25].

*ADRβ2* haplotype and haplotype pairs for each individual patient were inferred from the genotype data using the PHASE program. (v2.0). The PHASE prediction algorithm was run several times to ensure that the result was reproducible. The number and characteristics of haplotypes with frequency > 1% remained consistent. For each individual, only the most likely HP (predicted with over 80% accuracy) was considered in the final analysis.

### Statistical Analysis

Patient characteristics are expressed as mean with standard deviation (SD), unless otherwise stated. Demographic data and clinical parameters were compared between all groups using ANOVA and Tukey's test or where pooled data was used, Student's t-test for continuous data or with Chi square or Fishers' exact test (where appropriate) for categorical data.

Genotypic and allelic frequencies were calculated for each patient group stratified by asthma severity or atopy. Univariate analysis was then performed using chi-square tests and logistic regression analysis using SPSS v18.0 (IBM, Armonk, CA, USA). Power analysis showed that a difference of 10% in allelic and genotypic frequency could be detected, with 80% power and Bonferroni-corrected p-value of 0.05.

Frequency of the 5 most common HPs were calculated for mild, moderate and severe asthma group. The differences were tested using the odds ratio method where only a more stringent p value of 0.01 was considered statistically significant (Bonferroni-corrected). The prevalence of the HPs in the three asthma cohorts were also assessed using a logistic regression trend test performed on SAS v9.2 (SAS Institute Inc, Cary, CA, USA) to further support the association with asthma severity.

## Results

### Subjects

The overall demographic and clinical characteristics of the study cohort are summarised in Table 2. The LIWA cohort included a total of 883 unrelated Caucasians (125 severe, 172 moderate, 386 mild and 200 non-asthmatics) aged between 18 and 85. These four study cohorts were similar in age but there was a greater proportion of females (p <0.001) and a higher prevalence of atopy (p < 0.001) in the pooled asthmatic cohort compared with the non-asthmatics.

**Table 2:** Characteristics of Study Participants.

N = 2979	Non Asthmatic∧	Mild Asthma	Moderate Asthma	Severe Asthma
Patient (n)	2296	386	172	125
Age (years)	34.5 (16.6)§	49.1 (16.3)	50.0 (16.3)	54.0 (14.4)
Male (%)	41.2	36.5†	36.8	35.87†
Atopy (%)	52.0‡	82.1†	80.1	75.5†
Smoking History (pack yrs)	8.1 (14.9)‡	4.34 (4.9)†	4.36 (3.97)	4.99 (4.4)†
Mean Inhaled Steroid Dose^#^ (μg/day)	–	392.79 (105.2)	1248.0 (1052.42)	2208.16 (1327.70)¶
Steroid Resistance (%)	–	0	0	38.1
Daily Symptoms (times/week)**	–	3.11 (3.65)	7.37 (610)	11.68 (7.0)¶
SABA rescue therapy (times/week)**	–	3.14 (6.36)	10.9 (11.05)	17,8 (10.5)¶
Nocturnal Symptoms (times/week)**	–	0.70 (1.32)	1.18 (1.56)	3.69 (2.56)¶
Unplanned Doctor Visits (times/yr)*	–	0.40 (1.2)	1.03 (1.74)	2.80 (2.65)¶
Oral Steroid Use (times/year)*	–	0.09 (0.42)	0.35 (0.63)	4.4 (4.9)¶
Hospital Admissions (times/yr)*	–	0.03 (0.17)	0.07 (0.26)	1.28(2.39)¶
FEV_1_ (% predicted)	100.7 (17.0)‡	90.4 (18.02)	73.2 (22.15)	62.9 (23.6)¶
FVC (% predicted)	106.4 (16.9)‡	102.4 (16.8)	89.1 (19.5)	89.1 (19.5)¶
FEV_1_/FVC	77.4 (7.8)‡	73.9 (9.69)	68.4 (11.84)	64.0 (14.3)¶

∧Generated by pooling two cohorts one from the Queensland Institute of Medical Research (QIMR) and one from the Lung Institute of Western Australia (LIWA).

#Beclomethasone equivalent.

§ Only available in 63% (QIMR n = 1248, LIWA, n = 200), p<0.001 compared with other groups.

‡ Only available for the LIWA cohort (n = 200).

*Average number in the 12months prior to enrolment.

**Average number in the 3 months prior to enrolment.

† p value <0.001 comparing mild, moderate or severe asthmatic with non-asthmatic.

¶ p value <0.0001 comparing mild vs moderate, moderate vs severe and mild vs severe.

The severe, moderate and mild asthmatics were distinguishable in all clinical parameters including frequency of symptoms, lung function, medication use and health care utilisation (p<0.0001 for ANOVA and all subsequent pairwise analysis between severity groups, Table 2).

The QIMR_610K dataset (n = 2096) included 1248 non-asthmatics with mean age 31 (62% female) and 848 individuals (55.9% female, age unknown) with no available asthma information that were considered as unselected controls to improve power.[27]

### Genotype and Allelic Analysis

All participants were successfully genotyped for all SNPs (see Table 3). There was complete concordance between the Illumina and Taqman Assay results (n = 269). The allelic prevalence of *ADRβ2*+46*A and *ADRβ2*+79*C in the entire study cohort was 0.36 and 0.56, respectively. *ADRβ2*+491*T was only seen in the heterozygous form in 13 asthmatics (8 mild, 2 moderate, 4 severe) and 37 non-asthmatics. *ADRβ2* SNPs frequencies were comparable for all study groups and were within Hardy-Weinberg equilibrium (see Table 4). *ADRβ2* polymorphisms did not correlate with lung function for the whole group or when stratified by asthma severity (see Table 5). Despite lack of association between *ADRβ2* genotypes and asthma, there was a strong correlation between carriage of the C allele of SNP +523 and atopy (OR 2.05, CI 1.33-4.48 for +523CC or AC genotypes compared with +523AA, p = 0.03; Table 6).

**Table 3:** Genotypic and allelic frequency of *ADRβ2* polymorphisms stratified by asthma diagnosis and severity.

Polymorphisms (SNPs)	Non-Asthmatics (n = 2296)	Mild Asthma (n = 386)	Moderate Asthma (n = 172)	Severe Asthma (n = 125)
**−1023**				
Genotype (%)				
GG	32.8	30.9	30.1	27.9
GA	48.8	47.3	54.2	55.8
AA	18.4	21.8	15.7	16.3
Minor Allele (A) (%)	42.8	45.5	42.8	44.2
**−654**				
Genotype (%)				
GG	42.2	44.4	37.3	31.8
GA	45.1	41.0	51.6	55.6
AA	12.7	14.5	11.1	12.6
Minor Allele (A) (%)	35.3	34.9	36.9	37.1
**−468**				
Genotype (%)				
CC	34.0	31.3	30.1	27.4
CG	48.3	47.4	54.2	56.2
GG	17.7	21.5	15.7	28.6
Minor Allele (G) (%)	41.7	45.2	42.8	44.2
**−367**				
Genotype (%)				
CC	18.1	19.7	15.7	14.3
CT	48.5	49.0	54.2	57.1
TT	33.3	31.3	30.1	28.6
Minor Allele (C) (%)	42.4	44.2	34.5	42.9
**−47**				
Genotype (%)				
TT	33.3	31.3	30.1	28.6
TC	48.5	48.7	52.9	57.1
CC	18.1	19.9	17.0	14.3
Minor Allele (C) (%)	42.4	44.3	43.5	42.8
**−20**				
Genotype (%)				
TT	33.3	31.3	30.1	28.6
TC	48.5	48.7	52.9	57.1
CC	18.1	19.9	14.3	14.3
Minor Allele (C) (%)	42.4	44.3	43.5	42.8
**+46**				
Genotype (%)				
GG	43.1	44.3	37.9	35.4
GA	44.6	40.9	51.0	53.1
AA	12.3	14.8	14.8	11.6
Minor Allele (A) (%)	35.1	35.2	36.6	38.1
**+79**				
Genotype (%)				
CC	33.8	31.3	30.1	27.2
CG	48.5	47.4	52.9	56.5
GG	17.6	21.2	17.0	16.3
Minor Allele (G) (%)	42.5	44.9	43.6	44.6
**+252**				
Genotype (%)				
GG	60.8	64.2	64.7	64.7
GA	34.8	32.5	30.7	30.7
AA	4.4	3.4	4.6	4.6
Minor Allele (A) (%)	22.1	19.6	19.9	17.7
**+491**				
Genotype (%)				
CC	96.6	97.9	98.7	97.3
CT	3.4	2.1	1.3	2.7
TT	0.0	0.0	1.1	0.0
Minor Allele (A) (%)	1.7	1.0	0.6	1.4
**+523**				
Genotype (%)				
CC	65.2	66.8	66.0	72.8
CA	31.4	30.6	30.1	24.5
AA	3.4	2.6	3.9	2.7
Minor Allele (A) (%)	19.4	17.9	18.9	15.0

**Table 4:** Hardy Weinberg equilibrium value (χ2) for each ADRβ2 Polymorphisms.

SNP	χ2	P value
−1023	0.02	>0.05
−654	0.00	>0.05
−468	0.02	>0.05
−367	0.03	>0.05
−47	0.01	>0.05
−20	0.01	>0.05
+46	0.00	>0.05
+79	0.02	>0.05
+252	0.07	>0.05
+491	0.00	>0.05
+523	0.04	>0.05

**Table 5:** Lung function stratified by ADRβ2 genotypes.

SNP		Total‡		Mild Asthma		Moderate Asthma		Severe Asthma	
		FEV1*	FEV1/FVC	FEV1	FEV1/FVC	FEV1	FEV1/FVC	FEV1	FEV1/FVC
**−1023**	AA	85.9 (22.9)	72.3 (11.0)	91.4 (16.7)	74.1 (9.0)	73.0 (20.5)	72.4 (11.1)	59.9 (21.7)	63.8 (14.4)
	AG	84.5 (23.9)	72.2 (11.9)	88.9 (18.8)	73.6 (10.3)	76.1 (21.8)	66.7 (12.5)	61.6 (23.2)	63.7 (13.8)
	GG	86.1 (23.9)	73.1 (11.0)	91.6 (17.4)	74.4 (9.4)	68.8 (23.3)	69.1 (11.1)	64.5 (25.6)	65.0 (15.7)
**−654**	AA	85.8 (23.3)	73.0 (11.7)	91.9 (16.2)	73.8 (10.6)	67.7 (25.1)	70.3 (12.1)	66.6 (27.8)	66.3 (16.1)
	AG	85.5 (24.1)	72.5 (11.4)	91.4 (18.1)	74.4 (9.4)	75.5 (22.3)	67.2 (11.9)	62.3 (24.3)	63.6 (13.7)
	GG	86.0 (23.2)	72.8 (11.4)	89.1 (18.6)	73.5 (9.7)	71.9 (20.9)	69.3 (11.9)	61.0 (22.8)	64.1 (15.9)
**−486**	CC	86.4 (23.9)	73.3 (11.1)	92.0 (17.2)	74.6 (9.5)	68.8 (23.4)	69.1 (11.1)	63.5 (26.0)	64.4 (16.0)
	CG	84.6 (23.9)	72.3 (11.8)	88.9 (19.0)	73.6 (10.2)	76.1 (21.2)	66.7 (12.5)	62.2 (23.3)	64.3 (13.5)
	GG	85.7 (22.8)	72.1 (11.0)	91.4 (16.8)	73.9 (9.0)	73.0 (20.5)	72.4 (11.1)	59.9 (21.7)	63.8 (14.4)
**−367**	CC	85.8 (22.9)	72.2 (11.1)	90.9 (17.3)	73.8 (8.7)	73.0 (20.5)	72.4 (11.1)	59.7 (22.6)	63.1 (15.4)
	CT	84.8 (23.7)	72.4 (11.6)	89.1 (18.8)	73.5 (10.3)	76.1 (21.8)	66.7 (12.5)	62.4 (22.9)	64.6 (13.1)
	TT	86.2 (24.2)	73.0 (11.4)	92.0 (17.1)	74.7 (9.5)	68.8 (23.3)	69.1 (11.1)	62.6 (26.0)	63.5 (16.5)
**−47**	CC	85.8 (22.9)	72.2 (11.1)	91.1 (17.2)	73.8 (8.6)	72.9 (19.8)	72.4 11.1)	59.7 (22.6)	63.1 (!5.4)
	CT	84.8 (23.7)	72.4 (11.6)	89.0 (18.9)	73.5 (10.2)	76.2 (22.0)	66.7 (12.5)	62.4 (22.9)	64.6 (13.1)
	TT	86.2 (24.1)	73.0 (11.4)	92.0 (17.1)	74.7 (9.5)	68.8 (23.3)	69.1 (11.1)	62.6 (26.0)	63.5 (16.5)
**−20**	CC	85.8 (22.9)	72.2 (11.1)	91.1 (17.1)	73.8 (8.6)	72.9 (19.8)	72.4 (11.1)	59.7(22.6)	63.1 (15.4)
	CT	84.8 (23.7)	72.4 (11.6)	89.0 (18.9)	73.5 (10.3)	76.2 (22.0)	66.7 12.5)	62.4 (22.9)	64.6 (13.1)
	TT	86.2 (24.1)	73.0 (11.4)	92.0 (17.1)	74.7 (9.5)	68.8 (23.4)	69.1 (11.1)	62.6 (26.0)	63.5 (16.5)
**+46**	AA	86.2 (23.4)	73.3 (11.9)	92.7 (16.2)	74.6 (10.9)	67.7(25.1)	70.3 (12.1)	67.0 (28.3)	66.3 (16.4)
	AG	85.2 (24.2)	72.3 (11.4)	91.3 (17.9)	74.5 (9.5)	75.6 (22.6)	67.2 (11.9)	61.3 (23.7)	63.1 (13.2)
	GG	85.4 (23.2)	72.6 (11.4)	88.8 (18.6)	73.2 (9.5)	71.9 (20.6)	69.3 (11.9)	61.6 (22.0)	64.7 (15.2)
**+79**	CC	86.4 (23.9)	73.3 (11.1)	91.9 (17.1)	74.7 (9.5)	68.8 (23.3)	69.1 (11.1)	63.5 (26.0)	64.4 (16.0)
	CG	84.7 (23.9)	72.3 (11.9)	88.9 (19.0)	73.6 (10.2)	76.2 (22.0)	66.7 (12.5)	62.1 (23.2)	64.0 (13.7)
	GG	85.6 (22.8)	71.9 (10.9)	91.5 (16.9)	73.6 (8.9)	72.9 (19.8)	72.4 (11.1)	59.9 (21.7)	63.8 (14.4)
**+252**	AA	86.3 (22.8)	74.0 (9.5)	85.1 (19.5)	72.3 (9.4)	73.5 (26.4)	74.1 (8.16)	76.6 (23.6)	72.7 (19.2)
	AG	84.9 (24.3)	72.8 (11.4)	89.1 (19.3)	74.2 (9.4)	69.3 (20.7)	65.9 (12.2)	56.9 (20.8)	62.1 (13.9)
	GG	85.6 (23.5)	72.3 (11.6)	91.3 (17.3)	73.9 (9.9)	75.1 (22.5)	69.4 (11.9)	63.4 (11.9)	64.3 (14.2)
**+491**	CC	85.3 (23.7)	72.5 (11.4)	90.3 (18.0)	74.0 (9.8)	73.0 (22.1)	68.6 (11.7)	62.4 (23.7)	64.2 (14.2)
	CT	89.7 (23.4)	72.8 (12.9)	94.4 (19.1)	74.5 (7.6)	85.4 (26.2)	61.9 (10.8)	60.1 (12.6)	59.8 (16.7)
**+523**	AA	86.2 (24.3)	73.3 (106)	80.7 (19.7)	70.0 (9.4)	77.1 (27.0)	74.1 (18.2)	72.8 (25.4)	70.0 (18.5)
	AC	85.3 (24.2)	72.9 (11.6)	89.1 (19.7)	74.5 (9.5)	69.3 (21.1)	66.4 (12.2)	59.2 (22.3)	62.0 (15.1)
	CC	85.4 (23.5)	72.3 (11.4)	91.3 (17.1)	73.9 (9.8)	74.7 (22.3)	69.0 (11.9)	62.6 (24.0)	64.4 (13.9)

*values indicate % predicted.

‡ Lung function results only available for the LIWA cohort (n = 883).

**Table 6:** ADRβ2+523 Genotypes are Associated with Atopy (n = 2979).

SNP		Prevalence of Atopy (%)	Overall χ2 p value	OR for Pairwise Comparison*
**−1023**	**AA**	71.0	0.32	
	**AG**	76.5		
	**GG**	71.9		
**−654**	**AA**	72.6	0.50	
	**AG**	75.9		
	**GG**	71.7		
**−468**	**CC**	70.3	0.36	
	**CG**	76.1		
	**GG**	72.4		
**−367/−47/−20** **	**TT**	70.8	0.35	
	**CT**	75.9		
	**CC**	72.4		
**+46**	**AA**	73.5	0.79	
	**AG**	75.2		
	**GG**	72.6		
**+79**	**CC**	72.3	0.51	
	**CG**	76.0		
	**GG**	85.2		
**+252**	**AA**	64.7	0.19	
	**AG**	74.1		
	**CC**	74.3		
**+491**	**CC**	74.0	0.75	
	**CT**	71.4		
**+523**	**AA**	59.3	**0.06**	
	**AC**	74.0		OR 2.01 (1.29, 4.55), p = 0.0345
	**CC**	74.5		OR 2.04 (1.33, 4.55), p = 0.0344

*only tested against minor allele homozygotes if overall χ2 p value approximates statistical significance (0.05).

**Results the same for these 3 SNPs in tight linkage disequilibrium with inheritance of C or T alleles at every locus.

### Haplotype Analysis

The haplotype frequencies are summarised in Table 7. Nine of the ten Drysdale haplotypes [25] were identified with haplotype 2, 4 and 6 being the most common with frequencies of 43.9%, 34.5% and 15.0%, respectively. Two additional haplotypes were identified occurring at a frequency >1%, denoted as 2′ and 4′ as significant homology with Drysdale haplotype 2 and 4 existed. The differences consisted of three 5′UTR SNPs in linkage disequilibrium (D′≥0.97, *r* ^2^ = 1 *ADRβ2*-367, −47, 20) for 2′ and one 5′UTR SNP (*ADRβ2*-1023) for 4′. These two new haplotypes were found exclusively in asthmatics (see Table 4). While numbers were too small for further statistical analysis there was an overall trend for a higher frequency of pooled novel haplotypes in severe asthmatics compared with moderate, mild or non-asthmatics (p = 0.07).

**Table 7:** Haplotype frequencies stratified by asthma diagnosis and severity.

DH	*ADRβ2* SNPs											Frequency				
	−1023 (G/A)	−654 (G/A)	−468 (C/G)	−367 (T/C)	−47 (T/C)	−20 (T/C)	+46 (G/A)	+79 (C/G)	+252 (G/A)	+491 (C/T)	+523 (C/A)	Total	Non Asthmatic* (n = 2296)	Mild Asthma (n = 386)	Moderate Asthma (n = 172)	Severe Asthma (n = 125)
1	A	/	/	/	/	/	A	/	/	/	/	0.003	0.008	0.008	0.000	0.000
2	A	/	G	C	C	C	/	G	/	/	/	0.439	0.450	0.449	0.412	0.416
4	/	A	/	/	/	/	A	/	/	/	/	0.345	0.349	0.321	0.334	0.347
5	/	A	/	/	/	/	/	/	/	/	/	0.032	0.080	0.010	0.009	0.011
6	/	/	/	/	/	/	/	/	A	/	A	0.150	0.166	0.153	0.176	0.126
7	/	/	/	/	/	/	/	/	A	T	A	0.005	0.008	0.010	0.005	0.010
8	/	A	/	/	/	/	A	/	A	/	A	0.002	0.000	0.005	0.003	0.010
10	/	/	/	/	/	/	/	/	A	/	/	0.011	0.012	0.009	0.010	0.016
11	/	/	/	/	/	/	/	/	/	/	/	0.001	0.002	0.000	0.001	0.000
2′	A	/	G	/	/	/	/	G	/	/	/	0.003	0.000	0.018	0.009	0.026
4′	/	/	/	/	/	/	A	/	/	/	/	0.001	0.000	0.010	0.003	0.004

Abbreviations: DH, Drysdale Haplotypes.

*Includes pooling of cohorts from the Queensland Institute of Medical Research cohort and Lung Institute of Western Australia.

A dash ‘/’ represents the major allele.

2′ and 4’ represent haplotypes similar to DH 2 and 4, respectively, both occurred at frequency > 1%.

**Figure 2:**
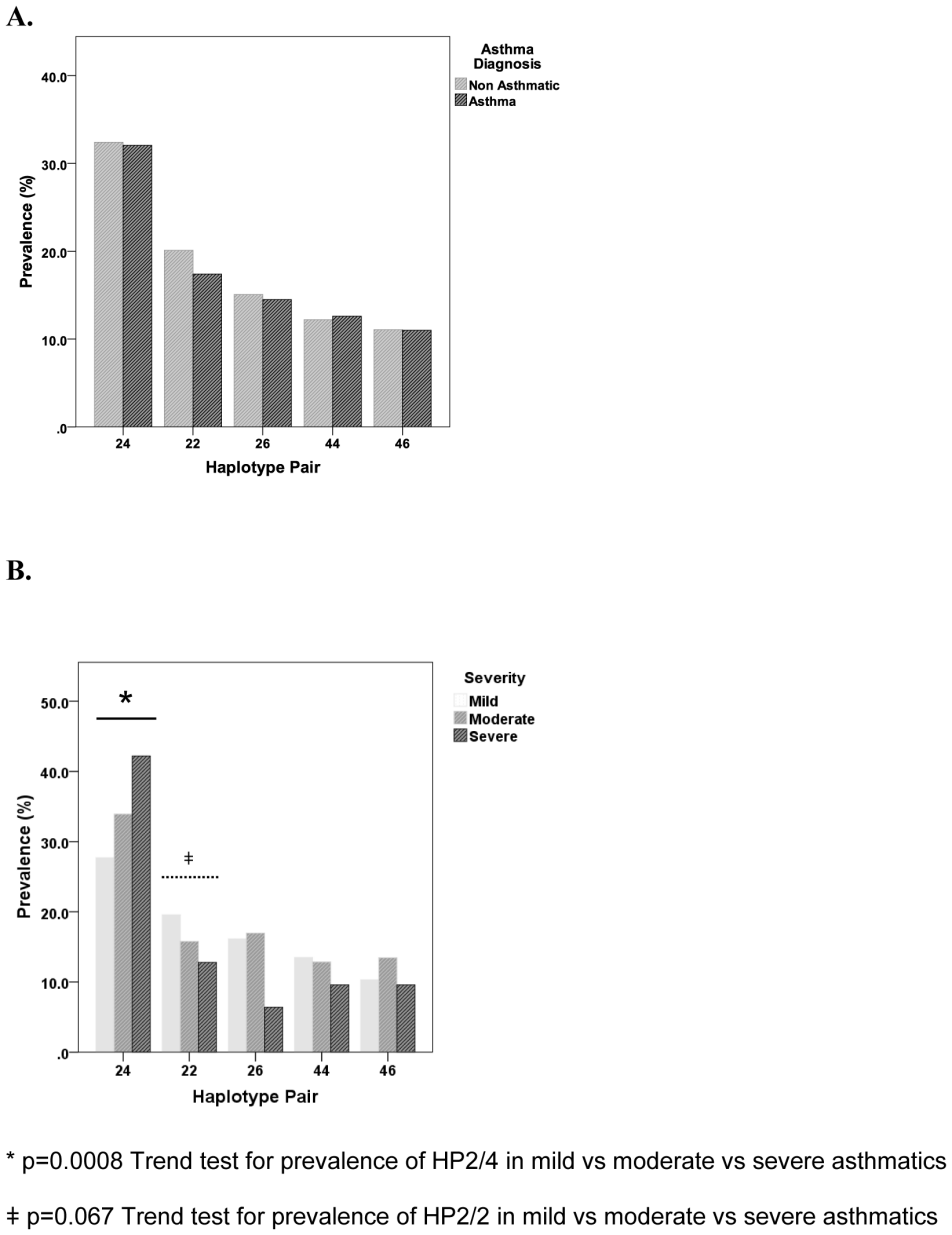
Comparison of prevalence of *ADRβ2* haplotype pairs with (A) asthma diagnosis (n = 2979) and (B) asthma severity (n = 683). The most common haplotype pair 2/4 was associated with severe asthma (Trend test, OR 1.42, p = 0.0008).

Overall, the most common HP was 2/4 (32.0%), followed by 2/2 (19.3%), 2/6 (14.8%), 4/4 (12.2%), 4/6 (10.9%) and 6/6 (3.0%). There was no association between any HPs and asthma diagnosis *per se* (Figure 2a). However, the frequency of HP2/4 increased with asthma severity (Figure 2b). The prevalence of HP2/4 was significantly higher in the severe asthma group (42.4%) compared with mild (27.7%, p = 0.0008 with OR 2.40, 95%CI 1.34, 3.11). The association was strengthened by a positive Trend test for mild, moderate and severe asthma (Mild OR 1.0; Moderate OR 1.42. 95%CI 1.16, 1.74; Severe OR 2.01, 95%CI 1.64, 2.46; p =  0.0008). Consequently, there was an absolute reduction in frequency of HP2/2 and 2/6, namely 6.8% and 9.7% in severe compared with mild asthmatics respectively. Although pairwise comparisons of the frequency of HP2/2 between mild, moderate and severe asthmatics were not significant, there was a trend for reduced prevalence with increasing asthma severity (Trend Test, OR 0.76, 95%CI 0.59, 1.02, p = 0.06, Figure 1b). In comparison, although the prevalence of HP2/6 in severe asthmatics was significantly different on pairwise comparison against mild or moderate asthmatics (OR 2.82, 95%CI 1.31, 6.06 p = 0.008; OR 2.98, 95%CI 1.32, 6.80, p = 0.008), there was no observable trend with asthma severity. The HP4/4 frequency was similar across the 3 cohort groups (14.5%, 13.2% and 11.6% for mild, moderate and severe asthmatics respectively, See Figure 1). There was no statistical difference in any clinical parameters when HP2/4 was compared with other HP for the asthmatic cohort as a whole or within each severity group. Within the severe asthma group, HP2/4 was not associated with steroid resistance.

**Figure 3:**
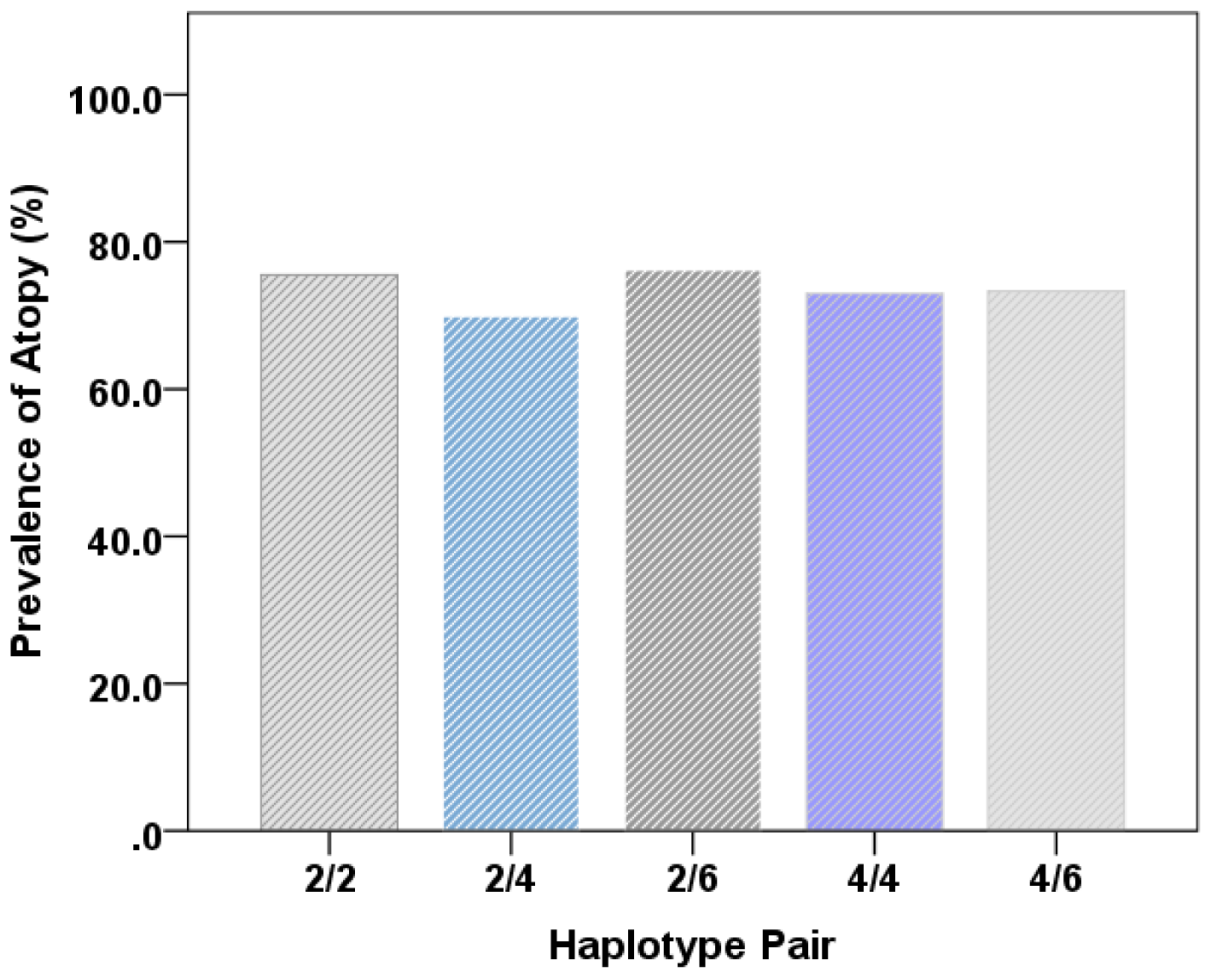
Prevalence of Atopy Similar Amongst Common ADRβ2 Haplotype Pairs (n = 2979).

The association with atopy was also present but not significantly strengthened in the relevant haplotype analysis inclusive of the +523CC genotype. Prevalence of atopy was also similar amongst the 6 most common haplotype pairs. (Figure 3).

## Discussion

This is the largest cohort of asthmatics and healthy controls to be studied in detail with respect to their *ADRβ2* haplotypic features. Given the heterogeneity in asthma pathogenesis and clinical manifestations, not unexpectedly, *ADRβ2* polymorphisms were not associated with asthma diagnosis or severity when individual SNPs or haplotypes were examined in isolation. However the frequency of the most common haplotype pair 2/4 was significantly higher in severe asthmatics with a raised odds ratio compared with mild asthmatics. The potential role for this *ADRβ2* haplotype pair in the pathogenesis of severe asthma was further supported by a significant, positive trend in the prevalence of haplotype pair 2/4 with increasing asthma severity. In addition, carriage of ADRβ2+523*C was associated with increased risk of atopy.

### ADRβ2 Haplotype Pair Is Associated With Asthma Severity

The haplotypes in this study were statistically inferred via the PHASE algorithm which is a well-recognised, time-efficient method for haplotype reconstruction using unphased genotype data derived from population samples such as ours. The accuracy and predictive power of PHASE algorithm was enhanced given our large sample size, the tight linkage disequilibrium between *ADRβ2* SNPs, successful genotyping for the entire cohort at each SNP locus and by performing the algorithm multiple times. In the vast majority of our cohort (99%), the final haplotype pair for each individual was predicted with over 98% specificity. Each of Drysdale's haplotypes are distinct and specifically defined by the subset of SNPs genotyped in this study.

Using a subset of 11 *ADRβ2* polymorphisms similar to those used by Drysdale *et al* [25], we identified nine Caucasian haplotypes whose frequency was similar to published data.[4], [25] Importantly two new haplotypes (2′ and 4′) were identified exclusively in asthmatics and possibly represent a rarer subset of Drysdale's haplotypes 2 and 4 contributing to asthma severity.

While previous studies have investigated the relationship between *ADRβ2* haplotypes and mild asthma [4], [25], [31], this is the first study to examine haplotypes and severe asthma. In agreement with previous studies, there was no association between single *ADRβ2* haplotypes and mild asthma. Five HPs (2/4, 2/2, 4/4, 2/6, 4/6) accounted for 80% of our subjects which is similar to that reported in mild or moderate asthma. [31] More importantly, HP2/4 was associated with severe asthma in the current study with an associated decreased frequency of HP2/2 and 2/6. Whilst it may be attractive to undertake a meta-analysis of our severe asthmatic subgroup and other studies on severe asthma [22]–[24], this is unfortunately unrealistic as these studies used varying definitions of asthma severity and genotyping was limited to only three *ADRβ2* SNPs.

The association between HP2/4 with asthma severity was unexpected but significantly substantiated by the findings from formal trend tests across the 3 asthma severity groups. It is worth noting that haplotype 2 and haplotype 4 are heterogeneous at the main functional locus as well as many others and it is therefore rather difficult to know how or which functional effects would be specifically driven when inherited as a haplotype pair. ADRβ2 preferentially exists as dimerised pair *in vivo*. Whether the coinheritance of haplotype 2 and 4 affects receptor dimerisation and therefore function of the combined receptor contributing to asthma pathogenesis needs to be considered and further explored.

There was no correlation between *ADRβ2* haplotypes and any individual clinical parameter used to define asthma severity. This is not surprising given that asthma is heterogeneous and classification of disease severity can be difficult when the various parameters used in defining severity are inter-related in many ways. In this study, there were no association between the inheritance of *ADRβ2* haplotypes and diagnosis of mild or moderate asthma. Inflammation plays a critical role in the pathogenesis of asthma but there is increasing evidence for considerable variability in the pattern of inflammation between individuals which most likely contributes to phenotypic differences in disease severity and treatment responses. Detailed examination of the role of *ADRβ2* haplotypes in the pathogenesis of asthma is beyond the scope of this study. However, it is unlikely any one gene can fully account for the pathogenesis of severe asthma but *ADRβ2* haplotypes may explain some of the therapeutic resistance seen in severe asthma. Mild asthmatics homozygous for *ADRβ2*+46*A bronchodilate to SABA[11] but have reduced PEFR and FEV_1_ and increased risk of exacerbation if SABA or LABA are used regularly.[13], [14], [16], [17] Contradictory findings on therapeutic responses in larger prospective studies [19]–[21] may be due to a lack of haplotypic analysis. The few studies that attempted to address the relationship between haplotypes and various clinical characteristics of asthma including pharmacological response in asthmatics are inconclusive. [32]–[37] Furthermore, the haplotypes in these analyses were simply defined by 2–4 SNPs which is often inadequate for more detailed distinction between *ADRβ2* haplotypes defined by Drysdale and colleagues.

Data on the effect of HPs on acute bronchodilator response (BDR) is limited and controversial. Taylor *et al* [31] showed no association between haplotype and BDR to inhaled salbutamol in mild to moderate asthmatics weaned off LABA, SABA and ICS. In contrast, Drysdale *et al* [25] reported a significant association between Drysdale haplotype two and a positive response to *ADRβ2* agonist. The response for HP2/4 was intermediate while HP4/4 showed a much weaker response. [25] Bronchodilator response was similarly high in HP2/6 and statistically no different to HP2/2. Whether Drysdale's *apriori* selection of patients with a positive BDR has led to selection bias is unclear. However, the implication that BDR can alter depending on prior treatment and HPs takes on much greater importance in severe asthma particularly with the current finding of a higher frequency of the less favourable HP2/4 and lower frequency of the more responsive HP2/2 and 2/6. [25] All of our asthmatics were selected based upon chronic disease status and not acute response to *ADRβ2* agonist and therefore similar to population studies by Drysdale *el al*. It is possible that the loss of protective, more responsive haplotypes 2 and 6 in severe asthmatics leads to a dynamic increase in the frequency of the most common haplotype pair in any given population, thus explaining the apparent association between HP2/4 and severe asthma.

To further enhance the current study data the functional significance of *ADRβ2* haplotypes with respect to *in vitro* receptor characteristics and response to different *ADRβ2* agonists needs addressing. Panebra *et al* reported variable baseline *ADRβ2* expression and agonist-induced downregulation between *ADRβ2* haplotypes. [38] Haplotype 2 demonstrated higher basal receptor expression while haplotype 4 showed greater downregulation following exposure to the *ADRβ2* agonists. Whether such observation translates to clinically important differences in acute bronchodilator response and tachyphylaxis respectively requires further investigation. Furthermore, the relative effect of each haplotype when coinherited as haplotype pair 2/4 is unknown. This would be best performed in a prospective pharmacogenetic study complementing *in vitro* studies to characterise the effect of haplotype pairs on receptor expression, function and regulation as well as pharmacological response. Such studies would provide important insights into whether individuals with severe asthma might benefit from alternative treatment strategies that minimise excessive use *ADRβ2* agonists.

### Association Between ADRβ2 +523CC Genotype and Atopy

The strong correlation between ADRβ2+523CC genotype and atopy is a novel finding. The precise mechanism for this is unclear and is beyond the scope of the current study. It is most likely that ADRβ2 +523C acts as a tag or proxy for the actual genetic variation associated with atopy. This would not be surprising given that the ADRβ2 gene is located on chromosome 5q, a region where multiple candidate gene polymorphisms has been associated with atopy and/or IgE production. These genes include interleukin 4 (IL4) [39], [40], interleukin 13 (IL13) [41], monocyte differentiation antigen CD14 [42]–[45] and serine protease inhibitor kazal 5 (SPINK5) [46], [47]. Unfortunately, there is no published data on whether polymorphisms of these candidate genes are in linkage disequilibrium with any of the ADRβ2 polymorphisms. Woszczek *et al* demonstrated significantly higher total IgE levels and prevalence of atopy in individuals homozygous with the ADRβ2 haplotype -47T/-20T/+46A/+79C/+252G. [48] Although their genotyping was not inclusive of SNP+523, results from the current study together with those reported in the literature [4], [25] suggest that the +523*C allele is likely to be in tight linkage equilibrium with the above haplotype.

ADRβ2+523C>A is a synonymous polymorphism in which both alleles produce the same protein without any changes in amino acid sequence. It was long assumed that synonymous SNPs are “silent” and inconsequential, as the primary sequence of the protein is retained. However, it has been shown in other disease states that synonymous polymorphisms can predict disease susceptibility if it is in linkage with another polymorphism of the same or different gene that contributes to disease pathogenesis. One such example is the association between the immune-related GTPase family M

(IRGM) gene and Crohn's inflammatory bowel disease. [49]–[52]

## Conclusions

Despite widespread use of *ADRβ2* agonists in the treatment of asthma, there is persistent background concern about their safety and efficacy. Functional polymorphisms of *ADRβ2* potentially generate differential treatment response and suboptimal outcomes for asthmatics treated with regular SABA or LABA. Most studies have investigated only a limited number of *ADRβ2* polymorphisms in mild or moderate asthmatics. However, it is the overall haplotype that best predicts receptor characteristics and treatment response. This study identified 13 new *ADRβ2* haplotypes and demonstrated, for the first time, that the *ADRβ2* haplotype pair 2/4 is associated with asthma severity. Further functional and clinical studies are needed to determine the role of *ADRβ2* polymorphisms/haplotypes both in the development of severe asthma and in influencing therapeutic responses to *ADRβ2* agonists. The positive association between ADRβ2+523CC genotype and atopy is novel. This most likely reflects its linkage with another candidate gene or causal polymorphism.
